# The Analysis of Genes and Phytohormone Metabolic Pathways Associated with Leaf Shape Development in *Liriodendron chinense* via De Novo Transcriptome Sequencing

**DOI:** 10.3390/genes9120577

**Published:** 2018-11-27

**Authors:** Jikai Ma, Lingmin Wei, Jiayu Li, Huogen Li

**Affiliations:** 1 College of Forestry, Nanjing Forestry University, Nanjing 210037, China; jikai0990@foxmail.com (J.M); Lingminw18@sina.cn (L.W.); lijiayuhp@foxmail.com (J.L.); 2 Co-Innovation Center for Sustainable Forestry in Southern China, Nanjing Forestry University, Nanjing 210037, China

**Keywords:** *Liriodronden chinense*, SEM, leaf development, RNA sequencing, DEGs

## Abstract

The leaf, a photosynthetic organ that plays an indispensable role in plant development and growth, has a certain ability to adapt to the environment and exhibits tremendous diversity among angiosperms. *Liriodendron chinense*, an ancestral angiosperm species, is very popular in landscaping. The leaf of this species has two lobes and resembles a Qing Dynasty Chinese robe; thus, leaf shape is the most valuable ornamental trait of the tree. In this work, to determine the candidate genes associated with leaf development in *L. chinense*, scanning electron microscopy (SEM) was employed to distinguish the developmental stages of tender leaves. Four stages were clearly separated, and transcriptome sequencing was performed for two special leaf stages. Altogether, there were 48.23 G clean reads in the libraries of the two leaf developmental stages, and 48,107 assembled unigenes were annotated with five databases. Among four libraries, 3118 differentially expressed genes (DEGs) were enriched in expression profiles. We selected ten DEGs associated with leaf development and validated their expression patterns via quantitative real-time PCR (qRT-PCR) assays. Most validation results were closely correlated with the RNA-sequencing data. Taken together, we examined the dynamic process of leaf development and indicated that several transcription factors and phytohormone metabolism genes may participate in leaf shape development. The transcriptome data analysis presented in this work aims to provide basic insights into the mechanisms mediating leaf development, and the results serve as a reference for the genetic breeding of ornamental traits in *L. chinense*.

## 1. Introduction

Since Darwin’s pioneering study, a long-standing challenge in biology has been the elucidation of the genetic basis of morphological evolution [1]. Leaf shape, a conspicuous angiosperm trait, presents enormous diversity and variability [2]. Despite this profound diversity in shape, leaf morphology is chiefly classified into simple and compound types. Both simple and compound leaves initiate from a pluripotent clustered cell of the shoot apical meristem (SAM) dome, a process followed by blade growth [3,4]. The margins of a blade, which can be smooth, lobed or serrated, mainly determine the shape of the leaf [5].

Previous evidence has demonstrated that leaf diversity, from the formation of the tiny SAM to lateral leaf outgrowth, is controlled by gene regulatory networks (GRNs) and signaling pathways [6] and there is a profusion of genes that play a central role in leaf initiation and outgrowth, including transcription factor (TF) family genes, such as *KNOTTED1-LIKE HOMEOBOX* (*KNOX*) [7,8,9], *CUP-SHAPED COTYLEDON* (*CUC*) [10,11], and *TEOSINTE BRANCHED 1-CYCLOIDEA-PCF* (*TCP*) [12,13], and phytohormone genes [13,14,15], such as *PINFORMD 1*(*PIN1*), *GA20-oxidase* (*GA20ox*) and *ISOPENTENYL TRANSFERASE 7* (*IPT7*). Beginning with the development of the leaf primordium, class I *KNOX* genes can regulate gibberellin (GA) and cytokinin (CK) biosynthesis, two phytohormone pathways that influence leaf initiation in the SAM [16,17]; this regulation is essential for SAM maintenance [8,18]. In tobacco, *NTH15* encoding the KNOX protein directly suppressed *NTC12*, a gene encoding a GA 20-oxidase enzyme, resulting in leaf morphological changes due to decreased levels of bioactive GA in the SAM [19]. Accordingly, *KNOX* gene expression levels have an effect on leaf shape, and it has been reported that the overexpression of *BREVIPEDICELLUS* (*BP*/*KNAT1*), a class I *KNOX* family gene, causes smooth leaves to become deeply lobed leaves in *Arabidopsis* [20]. Additionally, in transgenic strawberry, repressing or overexpressing *FaKNOX1* resulted in a conspicuous variation in leaf morphology [7].

In the past two decades, RNA-sequencing technology has emerged as a powerful tool to determine differentially expressed genes (DEGs) and the phytohormone signal transduction pathways in leaf development. For example, in *Gevuina avellana*, by using Illumina RNA-sequencing with large-scale transcriptome analysis, the DEGs of leaf primordia genes at different heteroblastic stages of responses has been enriched; these genes include *PIN1*, *Auxin Response Factor4* (*ARF4*) and *indole-3-acetic acid9* (*IAA9*), which regulate developmental programs controlled by hormones [21]. Moreover, in *Betula pendula*, in a comparison of the leaf transcriptome profiles between smooth and serrated leaves, a mass of DEGs were clustered, and the results showed impacts of these DEGs on leaf morphologic variation [22]. In addition, in an analysis of transcriptome data among domesticated tomato (*Solanum lycopersicum*) and two wild tomato relatives at four leaf developmental stages, a GRN for leaf development was identified. The results indicated the evolutionary transcriptomes and gene networks involved in leaf development and then characterized *BOP*, a *KNOX* family gene that participates in the establishment of leaf morphology and fruit maturity of tomato and repeatedly acts in leaf shape formation [1]. In summary, RNA-sequencing technology provides an approach that is applicable to deepening the understanding of organ development in plants.

As a versatile tree species, *Liriodendron chinense* is widespread in southern China and northern Vietnam [23]. On the one hand, this species is an excellent timber tree that is used to manufacture high-grade furniture. On the other hand, as a well-known tree in China, *L. chinense* is a popular ornamental plant in landscapes and courtyards. At present, the genus of *Liriodendron* contains only two species in nature: *L. chinense* and *Liriodendron tulipifera*. Moreover, *L. tulipifera* is the dominant species in North America, while its relative, *L. chinense*, is an endangered species that plays a critical role in phylogenetic studies of angiosperm in Eastern Asia. Usually, the leaves of the *Liriodendron* genus have two deep lobes on the leaf margin, which is the most valuable ornamental trait of the leaf and the trait that most differentiates *L. chinense* from *L. tulipifera*. In most cases, the *L. chinense* leaf has one deep lobe on each side, while there are 2–3 lobes on each side of the leaf in *L. tulipifera* [24]. Thus, the *L. chinense* leaf is an important representative material for the study of leaf shape development. Although many crucial genes regulating leaf shape have been well identified in a few angiosperms, such as Arabidopsis (*Arabidopsis thaliana*), tobacco (*Nicotiana tabacum*), maize (*Zea mays*), and tomato (*S. lycopersicum*), genes mediating leaf development in *L. chinense* remain unknown, with limited available genomic information. In this study, scanning electron microscopy (SEM) was employed to screen all the *L. chinense* leaves within a bud. Then, we obtained the transcriptomes for two stages of leaf development, conducted a further investigation of potential hormone signaling pathways, and validated the expression pattern of genes with respect to leaf developmental stages. Our findings not only present a potential genetic mechanism of leaf shape formation but also may provide a reference for ornamental breeding in *L. chinense*.

## 2. Materials and Methods

### 2.1. Plant Materials

The samples were collected from Xiashu, Jurong County, Jiangsu Province, China (119°13′20″ E, 32°7′8″ N), where 12 provenances of *L. chinense* from southern China were cultivated in 1994. In July, we sampled buds from the branches of the trees with a provenance of Wuyi Mountain, Fujian Province, China. Then, we examined the growth of dynamic tender leaves within the buds by microscopy to determine the leaf stages from which transcripts were obtained by RNA sequencing.

### 2.2. Scanning Electron Microscopy (SEM) Assay

The dissected buds were rinsed and stored overnight at 4 °C Formalin–acetic acid–alcohol (FAA) fixed liquid consisting of 38% formalin, glacial acetic acid and 50% alcohol. All the samples were then fixed in 1% OsO4 for 1 h. Before isoamyl acetate treatment, the fixed samples were gradually dehydrated with ethyl alcohol for 20 min at different concentrations (70%, 90%, 95% and 100%). Subsequently, the samples were dried with an EMITECH K850 critical point dryer (Emitech, Ashford, UK) and coated with an Edwards E-1010 ion sputter golden coater (Hitachi, Tokyo, Japan) and observed by FEI Quanta 200 FEG MKII scanning electron microscopy (FEI, Eindhoven, Netherlands) under a suitable pressure (1.94 × 10^−3^ Pa) at 10–20 KV of high voltage (HV).

### 2.3. Sample Preparation and RNA Extraction

According to the morphology of the samples, there were always 4–8 tender leaves within each bud, and we classified these leaves into Stages P1, P2, P3 and P4. However, because the samples in Stage P1 were too small to collect, approximately one hundred tender leaf samples from Stages P2, P3, and P4 were, respectively, collected in 1.5 mL tubes. Then, all the leaves of each stage were sampled and rapidly immersed in RNA Keeper Tissue Stabilizer (Vayzme, Nanjing, China) at 4 °C overnight. Total RNA was extracted from tender leaves using an RNA prep pure plant kit (Tiangen, Beijing, China) following the manufacturer’s instructions. The raw RNA sequencing data were deposited in the NCBI Sequence Read Archive (SRA) with the accession numbers SRR8101043, SRR8101042, SRR8101041 and SRR8101040.

### 2.4. cDNA Library Construction, RNA Sequencing and Assembly

In total, approximately 1 μg of qualified RNA per sample, with two biological replicates, was used as the input material for sequencing libraries. We used the VAHTS mRNA-seq v2 Library Prep Kit for Illumina (Vayzme, Nanjing, China) to construct four libraries: LcP2-1, LcP2-2, LcP7-1 and LcP7-2. Next, all raw data were obtained from the libraries via sequencing on an Illumina Hiseq X10 platform, which generated 150-bp paired-end reads. To obtain clean reads, the raw reads were cleaned by removing reads with adapter, ploy N and low-quality reads using SeqPrep (https://github.com/jstjohn/SeqPrep). The assembly was based on the clean data with high-quality analyses by using Trinity2.0 [25].

The mapped reads of each sample were assembled using Cufflinks (v2.2.1) [26] with a reference-based approach. We set Cufflinks in a probabilistic model, which provides a maximum likelihood explanation of the expression data in a given locus, to simultaneously assemble and quantify the expression patterns of a minimal set of isoforms. Then, to merge the assemblies of the samples, Cuffmerge was used to produce a master transcriptome, which was compared to known transcripts by Cuffcompare.

### 2.5. Identification of Differentially Expressed Genes (DEGs)

Cuffdiff (v2.2.1) provides statistical routines for determining differential expression in digital transcript or gene expression datasets using a model based on a negative binomial distribution. Transcripts or genes with corrected *p* values less than 0.05 and an absolute value of log2 (fold change) <1 were considered significantly differentially expressed.

### 2.6. Gene Ontology (GO) and KEGG Enrichment

The Gene Ontology (GO) enrichment analysis of DEGs was implemented with a Perl module (GO::TermFinder) [27,28]. GO terms with corrected *p* values less than 0.05 were regarded as significantly enriched among the DEGs. R functions (phyper and *q* value) were used to test for the statistical enrichment of the DEGs among the KEGG pathways. KEGG pathways with corrected *p* values less than 0.05 were considered significantly enriched among the DEGs.

### 2.7. Quantitative Real-Time PCR (qRT-PCR) Validation

To validate the transcriptome data profiles, ten DEGs with respect to leaf development were selected, and their expression patterns were identified via quantitative real-time PCR (qRT-PCR) with three biological replicates. Total RNA was extracted from the P2, P3 and P4 samples. Then, 300 ng of total RNA was reverse transcribed in a 20 μL reaction containing 5× PrimeScript RT Master Mix for Real time (TaKaRa, Shiga, Japan). All specific primers were 18–25 bp and generated 80–250 bp PCR products, and the Tm ranged from 58 to 60 °C. The primers are listed in Table S1. A reference housekeeping gene (β-Actin) was used for normalization. According to the manufacturer’s protocol, the reaction mixture was at a final volume of 20 μL consisting of 2 μL cDNA (diluted five times), 10 μL SYBR Premix Ex Taq (2×), 0.8 μL each specific primer (10 mM), 0.4 μL ROX Reference Dye II (50×), and 6.8 μL ddH2O (TaKaRa, Shiga, Japan). The thermal-cycler program was as follows: 60 s at 95 °C for the initial denaturation, followed by 40 cycles of 15 s at 95 °C and 60 s at 60 °C, and 30 s at 60 °C for annealing.

## 3. Results

### 3.1. Dynamic Development of Leaves in *L. chinense*

**Figure 1:**
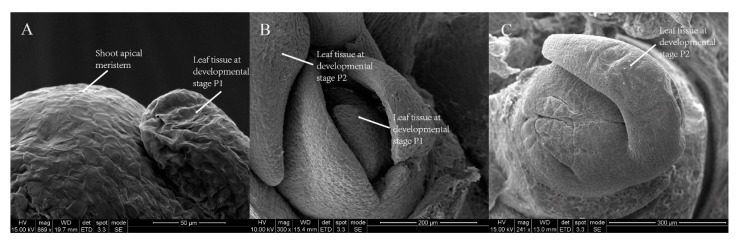

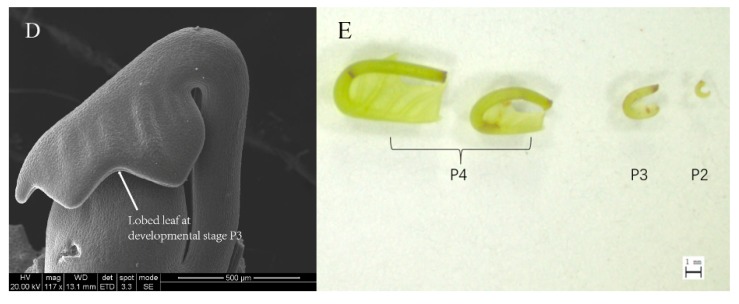
The dynamic development of *L. chinense* leaves. (**A**–**D**) Leaves in different bud layers: (**A**) a leaf tissue at developmental Stage P1 and shoot apical meristem (SAM) dome; (**B**) a leaf tissue at developmental Stage P1 and a leaf tissue at developmental Stage P2; (**C**) a leaf tissue taking the shape of a small fishhook at Stage P2; and (**D**) the first lobed leaf at Stage P3. (**E**) Four samples of leaves at Stages P2, P3 and P4 within a bud. Scale bars: 50 μm (**A**); 200 μm (**B**); 300 μm (**C**); 500 μm (**D**); and 1 mm (**E**). The abbreviations in the scanning electron microscopy (SEM) figures are defined in Table S4.

To determine where and when leaf organs arise, all leaves within a bud were sampled and screened. According to the developmental morphology of the samples, the leaves within a bud can be separated into four stages: P1, P2, P3 and P4. Likewise, we considered that the leaf developmental process includes three pivotal events: initiation, outgrowth, and expansion. First, we screened a tissue-like leaf primordium at an early recognizable stage of leaf development; initiating from the flank of the SAM dome, it was approximately 50 μm in size, and the SAM diameter was only approximately 100 μm in the final layer of the bud at this stage (Figure 1A,B). Later, tissue with a size of 200–300 μm took the shape of a small fishhook and differentiated into the leaf blade and petiole in the second layer of the bud at developmental Stage P2 (Figure 1C). Notably, at Stage P3, we found the first lobed leaf with sculptured veins on the back of the blade in a bud (Figure 1D). At Stage P4, the leaf lobe was deeper than that at Stage P3, and the prototype of the leaf morphology was achieved (Figure 1E).

### 3.2. Transcriptome Sequencing and De Novo Assembly

In total, we generated 92,146,944 raw reads from Lcp2-1, 101,976,354 raw reads from Lcp7-1, 88,182,826 raw reads from Lcp2-2 and 106,177,106 raw reads from Lcp7-2 with 47.98%, 48.84%, 47.67% and 48.53% GC contents, respectively (Table 1). After filtering the low-quality data, we obtained 385,866,426 clean reads for de novo assembly. Furthermore, all the transcripts were clustered into 80,492 unigenes with a mean length of 1025 bp and an N50 value of 1778. Among all the unigenes, 11,720 (14.56%) unigenes were more than 2000 bp in length, and transcripts with lengths between 100 and 500 bp were generated in 36,100 (48.85%) of the unigenes (Table 2).

### 3.3. Functional Annotation and Classification

**Figure 2:**
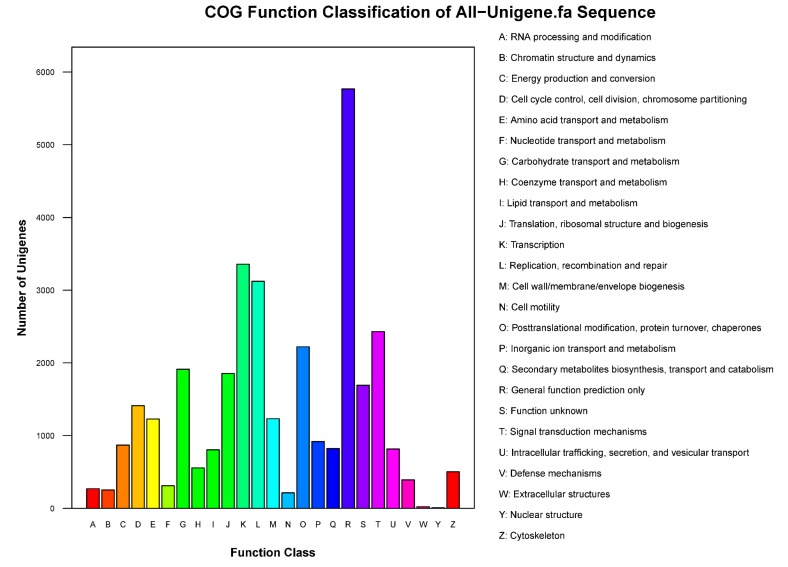
Clusters of orthologous groups of proteins (COG) function classification of unigenes. The vertical axis indicates the number of unigenes, and the horizontal axis indicates the function class of the unigenes.

In the clusters of orthologous groups of proteins (COG) functional classification plot, 74,766 unigenes were classified into 26 groups. Moreover, the R function class (general function prediction only) contained approximately 5767 unigenes, and the J function class (translation, ribosomal structure and biogenesis) contained 3357 unigenes (Figure 2). All unigenes with an average length of 730 bp were annotated according to the Nr (44,244 91.97%), Nt (38,131 79.26%), Swiss-Prot (28,673 59.60%), KEGG (27,455 57.07%), COG (17,318 36.00%) and GO (31,666 65.82%) databases (Table 3).

### 3.4. Gene Ontology (GO) Classification of Annotated Unigenes

**Figure 3:**
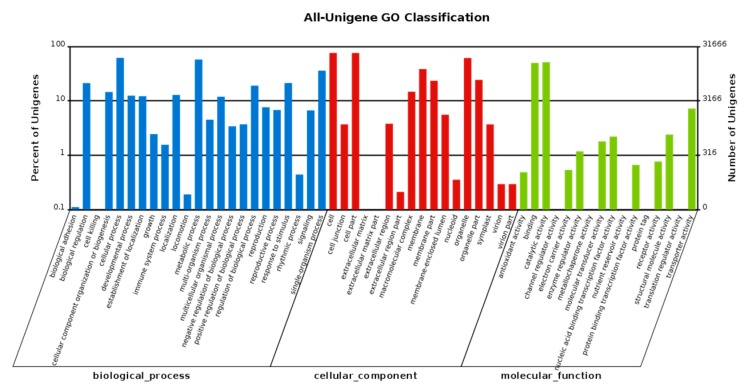
GO classification of unigenes. The horizontal axis shows the GO functional types, and the right-side vertical axis represents the total number of annotated unigenes, while the left-side vertical axis represents the percentage of the unigenes.

Gene ontology (GO), a dynamically updated vocabulary describing the characteristics of genes and genetic products in organisms, is an international standardization of the gene function classification system. GO classification is used to classify the unigene functions based on the Nr annotation results. There are three main categories of GO: “molecular function”, “cellular component” and “biological process”. In the biological process category, “cellular process”, with 19,541 unigenes, was the largest subcategory. In the cellular component category, “cell”, with 24,075 unigenes, was the largest subcategory and was the most annotated process overall. Most of the “molecular function” unigenes were classified in the catalytic activity subcategory, with 16,258 unigenes, followed by the binding group subcategory, with 15,762 unigenes (Figure 3).

### 3.5. Differentially Expressed Gene (DEG) Identification and Phytohormone Metabolic Analysis

**Figure 4:**
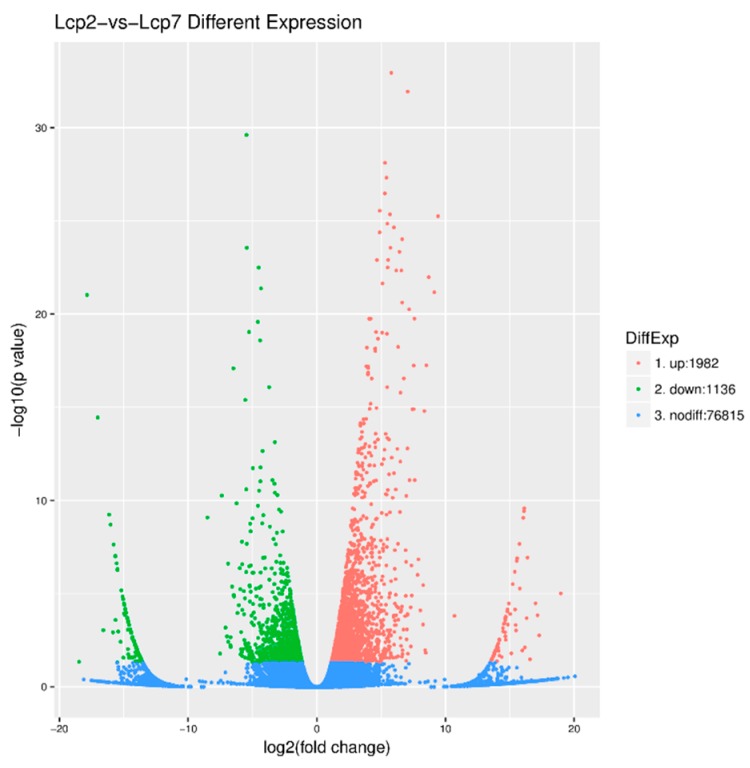
Differentially expressed genes (DEGs) between two leaf developmental stages.

Setting the fold change ≥2.0 and *p* < 0.05, we generated 3118 DEGs and 76,815 non-differentially expressed genes. The details of the 3118 DEGs are listed in Table S3. A total of 1982 DEGs, which are indicated by red dots in Figure 4, were up-regulated. Similarly, the green dots in Figure 4 represent the 1136 DEGs that were down-regulated, and the profusion of blue dots represents genes with no differential expression (Figure 4).

**Figure 5:**
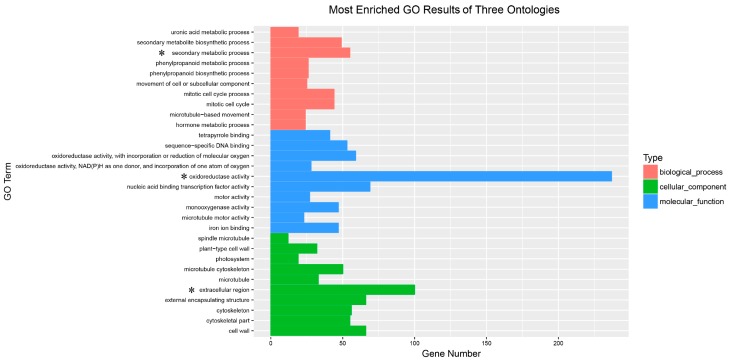
The most enriched GO results of three ontologies. The significant category in each of these three ontologies is marked with an asterisk.

Based on the GO enrichment results, we plotted the top ten terms in each GO category. Apparently, “oxidoreductase activity” was enriched by the most DEGs (237 DEGs) and was the most represented term in the molecular function category. In addition, the most DEG-enriched term in the cellular category was “extracellular region”, which contained 100 DEGs. There were four outstanding terms in the biological process category: “secondary metabolite process” (55), “secondary metabolite biosynthetic process” (49), “mitotic cell cycle process” (44) and “mitotic cell cycle” (44) (Figure 5).

**Figure 6:**
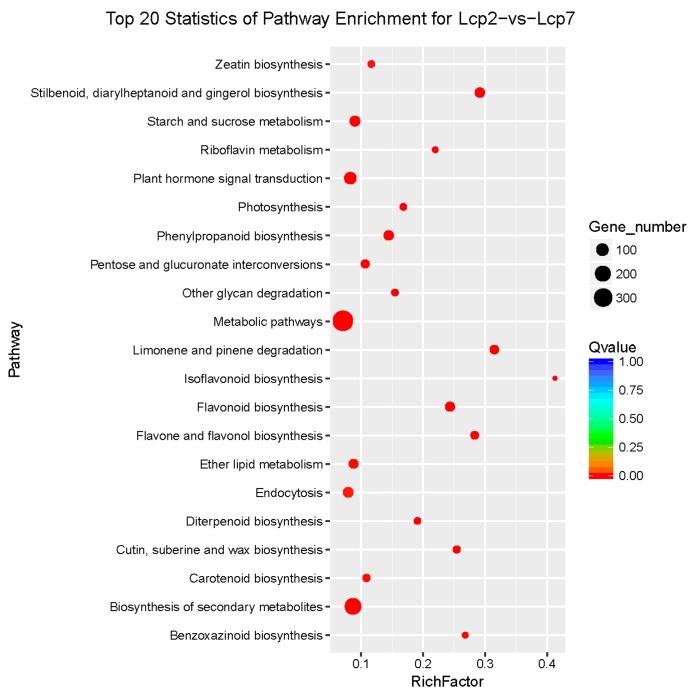
KEGG pathway enrichment scatter distribution. The rich factor is the ratio of DEGs in a pathway to all annotated genes in the pathway. The *q* value is the *p* value corrected by the multiple hypothesis correction test. Enrichments are significant when the *q* value approaches zero.

The enrichment level of KEGG pathways was measured by the rich factor, *q* value and enriched gene number. The plot in Figure 6 shows the twenty most significant pathways, based on the above criteria, enriched in a comparison of the two studied leaf developmental stages. To better characterize the biological functions in leaf development, the DEGs from the two studied stages of leaf development were further analyzed with the KEGG database. In this analysis, as shown in Figure 6, the red dots indicate that the metabolic pathways were enriched with more than 300 DEGs, which indicated the active metabolism of leaf development (Figure 6). However, we mainly focused on the plant hormone signal transduction pathway, which was significantly enriched by approximately 100 DEGs. These enrichments were significant and reliable, and thus, all the dots in the pathway plot are red.

**Figure 7:**
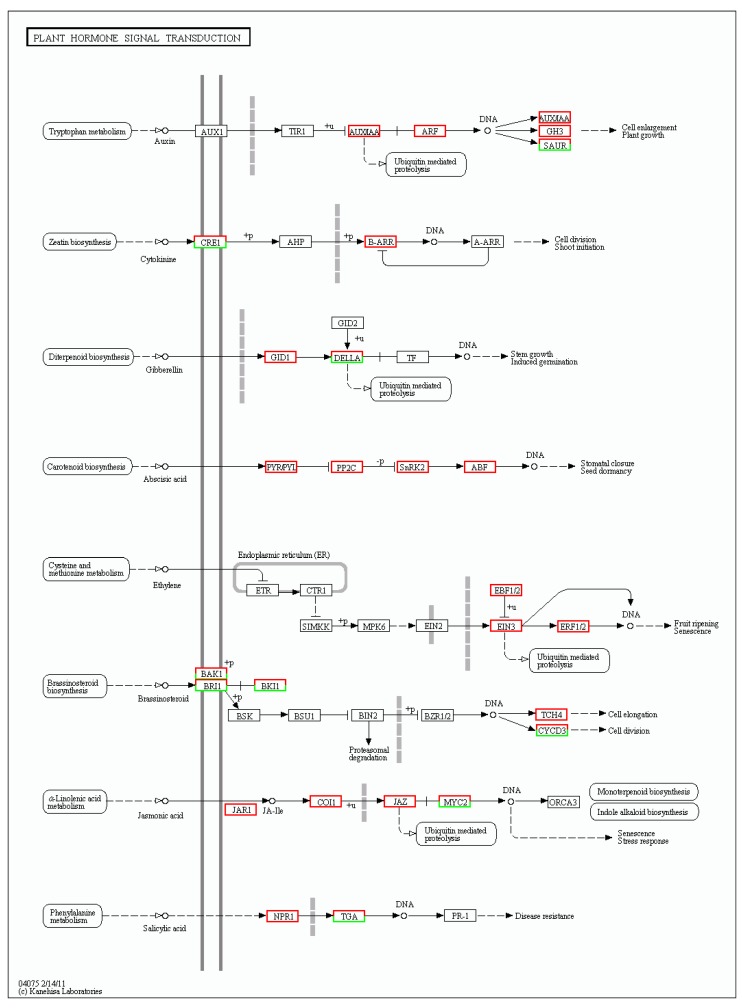
DEGs involved in the plant hormone signal transduction pathway. The enriched KEGG ortholog (KO) database terms are colored according to DEG regulation: red represents up-regulation, while green means down-regulation.

Plant hormone signal transduction significantly plays a vital role in plant morphological establishment. In Figure 7, which compares two stages of leaf development, there are obviously more up-regulated genes than down-regulated genes in the seven plant hormone signal transduction pathways: auxin, cytokinin, abscisic acid, ethylene, brassinosteroid, jasmonic acid and salicylic acid. To clearly visualize differential expression in the hormone signal transduction pathways, the upregulated genes in subpathways are shown in red boxes, and the downregulated genes in subpathways are shown in green boxes. Clearly, the auxin metabolic pathway was very active, and five boxes were up-regulated, including twenty-three up-regulated genes and only one down-regulated gene in the auxin metabolic pathway. Additionally, there were seven down-regulated genes and three up-regulated genes in the CRE1 box and three up-regulated genes in the B-ARR box, which participate in the cytokinin metabolic pathway. In the gibberellin pathway, two boxes had DEGs composed of six up-regulated genes and one down-regulated gene. Those three phytohormone pathways were the most important metabolic pathways on which we mainly focused. In the abscisic acid and ethylene pathways, all DEGs were mediated in four boxes and three boxes, respectively. In the brassinosteroid pathway, four boxes had both up-regulated DEGs and down-regulated DEGs, and there was only one up-regulated DEG in the TCH4 box. Furthermore, there were seven up-regulated DEGs and four down-regulated DEGs in the jasmonic acid pathway. In the salicylic acid pathway, there were four up-regulated DEGs and two down-regulated DEGs in the TAG box, while only one DEG was present in the NPR1 box (Figure 7). These results showed a profusion of up-regulated genes in metabolic pathways and indicated positive phytohormone action during the leaf development process.

### 3.6. qRT-PCR Validation of Differentially Expressed Geness (DEGs) Associated with Leaf Development

**Figure 8:**
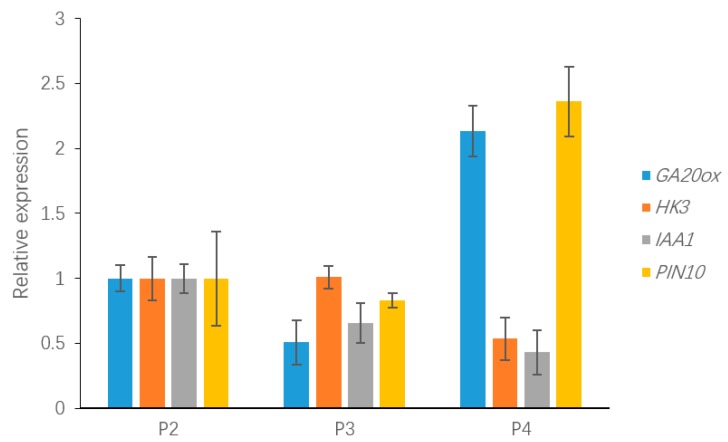
The expression of DEGs related to plant hormone signal transduction, including *GA20ox*, *PIN10*, *IAA1* and *HISTINDINE KINASE3*, which may be involved in leaf development.

**Figure 9:**
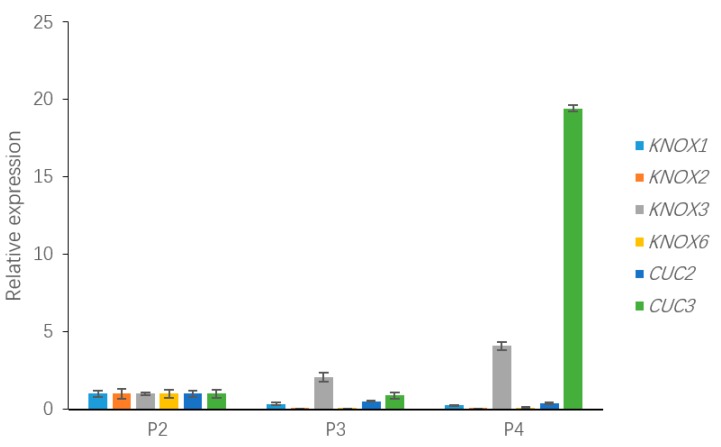
The expression of DEGs in the *KNOTTED1-LIKE HOMEOBOX* family and *CUP-SHAPED COTYLEDON* TF family, including *KNOX1*, *KNOX2*, *KNOX3*, *KNOX6*, *CUC2*, and *CUC3*, which may be involved in leaf development.

**Figure 10:**
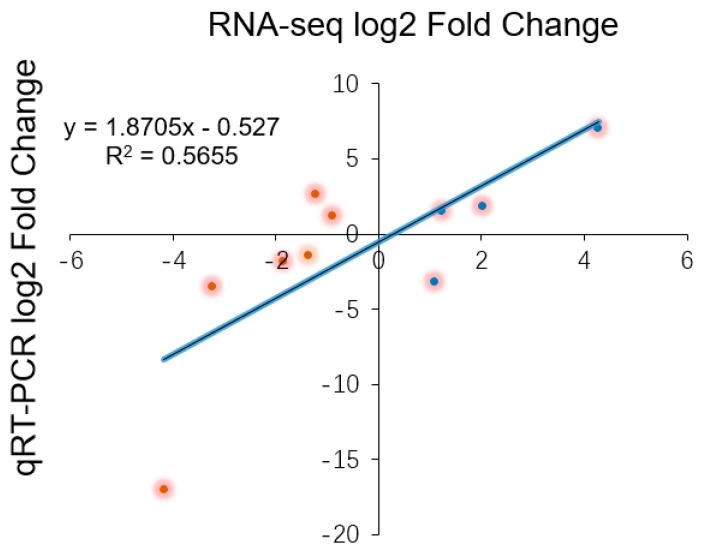
The logarithmic fold change values for the RNA-sequencing and qRT-PCR results are plotted along with the linear fit line to identify the correlation between two techniques (R^2^ = 0.5655, *p* < 0.01).

To validate the relative expression levels from the transcript abundance estimation, we selected ten DEGs related to leaf growth regulation: *GA20ox*, *PIN10*, *IAA1*, *HK3*, *KNOX1*, *KNOX2*, *KNOX3*, *KNOX6*, *CUC2*, and *CUC3* (Table S2). Of those, the GA20ox protein can directly repress the gibberellin level [29,30]. *IAA1* and *PINFORMD10* (*PIN10*) are genes influencing the auxin level [31]; *HK3* (*HISTIDINE KINASE3*) mediates the cytokinin level [32]; and *KNOX2*, *KNOX3* and *KNOX6* are TFs encoding knotted-like homeobox family proteins. The expression levels of all of these DEGs were associated with transcriptome analysis. Four of the DEGs—*GA20ox*, *PIN10*, *IAA1*, *HK3—*are plant hormone signal transduction factors, and the remaining DEGs, mainly consisting of *KNOTTED1-LIKE HOMEOBOX* TF family genes [33,34] and *CUP-SHAPED COTYLEDON* TF family genes [11,35], are closely related to the regulation of leaf growth. Plant hormones have a great effect on leaf initiation and outgrowth. *IAA1* and *PIN10* are related to the auxin hormone metabolism pathway, but the expression of these genes exhibited different trends in this work. Indeed, *PIN10* expression levels were lower in Stage P2 than in Stage P4, but *IAA1* showed a trend of down-regulation during three stages. In addition, one of the GA pathway genes and one of the CK pathway genes were used to verify the reliability of the transcriptome data and showed a correlation with the transcriptome results (Figure 8). According to a previous study, class I *KNOTTED1-LIKE HOMEOBOX* family genes mainly express in the SAM and primordium tissue. In this work, the *KNOX6* gene exhibited low expression levels at leaf developmental Stages P3 and P4 but had high expression levels at Stage P2. Furthermore, *KNOX3* showed a trend of up-regulated expression during three stages. Actually, *KNOX3* belongs to the class II *KNOTTED1-LIKE HOMEOBOX* family, and the main functions of this subfamily are barely related to the establishment of leaf morphology. The *CUC3* gene exhibited a very high expression level in Stage P4 but was seldom expressed in Stages P2 and P3. Additionally, the expression levels of *KNOX2* exhibited a decreasing trend while the *KNOX3* gene showed an increasing expression trend (Figure 9). The expression levels of ten selected genes as measured by RNA-sequencing and qRT-PCR were correlated. Most of the points were close to the line and the *p*-value was <0.01; R^2^ = 0.5655 (Figure 10). Taken together, the transcript data were reliable and reproducible; most of the qRT-PCR validation results were correlated with the DEG data.

## 4. Discussion

### 4.1. Leaf Initiation and Dynamic Development

To achieve their final shape and size, plant leaves must undergo three pivotal events: initiation, outgrowth, and expansion. Regardless of the final leaf shape, the incipient leaf initiates as a simple primordium from the SAM [36]. Once asymmetry has formed, the leaf primordium, initiated from the SAM, undergoes elongation and partitioning into a proximal petiole [5]. For this study, we aimed to examine the dynamic process of leaf development in *L. chinense*. Using the SEM method, we classified all leaves within a bud into Stages P1, P2, P3 and P4. In *Arabidopsis*, to achieve the final leaf morphology, leaf development is followed by the progression of trichomes [37], the provascular strand [38], enlarged epidermal cells [39], modified cellular morphology, and differentiating guard cells [40]. We captured the image of tissue-like leaf primordium with only 50 μm size at Stage P1. After that, the tissue differentiated into leaf blade and petiole. Furthermore, the first lobed leaf, composed of a petiole, lamina and leaf lateral vein, arose at Stage P3. Subsequently, the morphology of the leaf steadily developed in Stage P4. Overall, since the leaf obviously differentiated into blade and petiole at Stage P2 and leaf shape was achieved at Stage P4, samples from these two special stages were analyzed with transcriptome sequencing.

### 4.2. Genes Associated with Leaf Development

Hitherto, many works have identified the genes regulating leaf shape and variation, such as *CUC2/3*, *CIN* and *PIN*, that have significant effects on the processes of leaf development [41]. In this work, among the 3118 identified DEGs, ten DEGs associated with leaf shape in several model plants, such as the *KNOTTED1-LIKE HOMEOBOX* family genes, the *CUP-SHAPED COTYLEDON* family genes and *IAA1*, *GA20ox* and *HISTIDINE KINASE3* genes from the auxin, gibberellin and cytokinin hormone transduction signal pathways, were selected to validate the relative expression levels by qRT-PCR assays. The differential regulation and descriptions of these genes are listed in Table S2. 

The expression of the “meristem” genes in the leaf primordium could affect leaf shape and size via regulating hormone gradients and possibly transport [3]. A large body of evidence suggests that the precise regulation of *KNOX* gene activity is crucial to the determination of organ versus meristem identity in many plant species [14]. According to the annotation results, five DEGs were annotated with the *KNOTTED1-LIKE HOMEOBOX* family term, and the expression levels of four of these candidate DEGs were validated. Notably, the *KNOX2* gene was expressed at very low levels in Stages P3 and P4 but had a high expression level in Stage P2. A similar situation has been reported in *Arabidopsis*, in which the *KNOX* family consists of three classes: class I, class II and class III. Class I contains *SHOOTMERISTEMLESS* (*STM*), *BREVIPEDICELLUS* (*BP*/*KNAT1*), *KNAT2* and *KNAT6*, four genes with specific expression in the plant meristem [42]. All of these genes have a conserved homeodomain (HD) encoding the HD protein [43]. In addition, previous studies have concentrated on the role that hormones, such as auxin, play in releasing biophysical constraints on the incipient primordium and have indicated that the final stage of leaf formation is supported by the coordination of hormonally mediated cell processes, division and differentiation [3]. According to the KEGG enrichment analysis, approximately 100 DEGs with rich factors approaching 0.1 were clustered in the plant hormone signal transduction pathway. Of these, from the gibberellin and auxin biosynthesis metabolic pathways, we obtained several key DEGs that may participate in leaf shape development. Moreover, our results also showed that the *KNOX1* gene, which was mainly up-regulated in Stage P2, showed a low expression level and down-regulation in developmental Stages P3 and P4. Furthermore, the *KNOX2* and *KNOX6* genes showed extremely low expression patterns in developmental Stages P3 and P4. According to previous work, the *NTH15* gene encoding the tobacco KNOX protein, directly binds to GTGAC, an intron sequence of *NTC12* encoding GA20-oxidase, which is essential for the biosynthesis of gibberellin, resulting in a decrease in GA levels, and this decrease may influence cell proliferation and cell differentiation [19]. Coincidentally, in our validation analysis, the *GA20ox* gene expression level demonstrated a certain correlation with *KNOX2* gene expression in Stages P2 and P3. In several plants, due to the downregulation of *KNOX1* in different pathways, low cytokinin to high gibberellin levels are attained, and pathways are regulated by auxin via polar transport at the sites of leaf initiation, thereby repressing *KNOX1* [44] and cytokinin signaling at specific districts [45].

Previous work investigating a wide range of lobed/serrated leaves has shown that *CUC2* is a dispensable factor [11]. Several works suggest that *CUC2* expression is repressed by *PIN1*-generated auxin maxima [46]. In addition, *CUC2* is always down-regulated at the leaf margin, where *PIN1* convergent polarities are expected to exhibit high auxin levels [47,48]. Our observations suggested that *CUC2* expression levels were high at Stage P4, whereas *PIN10*, encoding an auxin efflux protein [49], synchronously showed low expression levels. *CUC2* is considered to affect the early onset of leaf teeth, but *CUC3* is thought to take part in sustaining leaf teeth outgrowth only at later stages [46]. However, there was no direct relationship between *CUC2* and *CUC3* in the validation data. Furthermore, the *PIN10* expression trend did not exhibit a correlation with the *IAA1* trend, even though the *IAA* family is also closely associated with the auxin hormone level [15]. Altogether, we indicated a few genes that are related to leaf development, such as the *KNOX2*, *KNOX6* and *CUC2* genes, and these findings may provide insights into the mechanisms mediating leaf development in *L. chinense*.

## 5. Conclusions

In summary, we classified all the developing tender leaves within a bud into Stages P1, P2, P3 and P4, and two special stages of leaves were analyzed by transcriptome sequencing. In this work, 385,866,426 clean reads were assembled into 298,261 unigenes, with a mean length of 732 bp. Of these, 158,814 (53.25%) unigenes were annotated to the Nr, Nt, SWISS, COG and KEGG databases, which are five publicly available protein databases with significant similarity. Moreover, we obtained 3118 DEGs, ten of which were selected as candidate DEGs. These candidate DEGs related to leaf development were validated in the four leaf stages, and most transcriptome data were closely correlated with qRT-PCR results. Consequently, these findings may provide insight into the mechanisms mediating leaf development and a reference for genetic breeding of ornamental characteristics in *L. chinense*.

## Figures and Tables

**Table 1:** Read quality and statistics.

Samples	Total Raw Reads	Total Clean Reads	Total Clean Nucleotides (nt)	Q20 Percentage	Q30 Percentage	N Percentage	GC Percentage
LcP2-1	92,146,944	91,485,392	13,722,808,800	97.09%	93.07%	0.00%	47.98%
LcP7-1	101,976,354	101,267,942	15,190,191,300	97.06%	93.02%	0.00%	48.84%
LcP2-2	88,182,826	87,727,852	13,159,177,800	97.03%	92.95%	0.00%	47.67%
LcP7-2	106,177,106	105,385,240	15,807,786,000	96.97%	92.83%	0.00%	48.53%
Summary	388,483,230	385,866,426	57,879,963,900				

Q30 indicates the nucleotides with quality values ≥30, and N percentage is the proportion of unknown nucleotides in the clean reads. The GC percentage is the proportion of guanidine and cytosine among all the nucleotides. Lcp2-1 and Lcp2-2 are two replicates of the same stage.

**Table 2:** Assembly and quality of unigenes.

Unigene Length	Total Number	Percentage
100–500 bp	36,100	44.85%
500–1000 bp	15,876	19.72%
1000–1500 bp	9746	12.11%
1500–2000 bp	7050	8.76%
≥2000 bp	11,720	14.56%
N50	1778	
Mean	1025	
All unigenes	80,492	
Length of all unigenes (bp)	82,472,451	

**Table 3:** Unigenes annotated with six databases.

Database	Total Unigenes	Annotated Unigenes	Percentage of Annotated Unigenes
Nr	48,107	44,244	91.97%
Nt		38,131	79.26%
Swiss-Prot		28,673	59.60%
KEGG		27,455	57.07%
COG		17,318	36.00%
GO		31,666	65.82%

## Supplementary Materials

The following are available online at http://www.mdpi.com/2073-4425/9/12/577/s1, Table S1: All the qRT-PCR primer sequences, Table S2: Annotation of candidate genes related to leaf development, Table S3: The list of DEGs, Table S4: The abbreviations in text.
