# Supplementary material for: The Analysis of Genes and Phytohormone Metabolic Pathways Associated with Leaf Shape Development in Liriodendron chinense via De Novo Transcriptome Sequencing
Source: Genes (Basel). 2018 Nov 27;9(12):577. doi: 10.3390/genes9120577 (PMC6316054; doi:10.3390/genes9120577)
Supplement: Supplementary file 1 [file genes-09-00577-s001.zip › 10.genes-366719-supplementary/Table S1. All qRT-PCR primer sequences.docx]

**Table S1.** All qRT-PCR primer sequences.

| Annotation name | Primer sequences | Tm (℃) |
| --- | --- | --- |
| *KNOX 1* | TCAAGATCATCGACGGCAGCA | 60 |
|  | GCTCCGATTTCTCCACAAACCC | 60 |
| *KNOX 2* | ACTACCTGAGATTGATCCTCGT | 60 |
|  | GCTCCCACCAGTTGAGTAGC | 60 |
| *KNOX 3* | TGAATGAAGCCTCGCCGAAC | 60 |
|  | CACAACATTGGCTATGCACGAA | 60 |
| *KNOX 6* | CCAAGAAATTGCACCGCGAG | 60 |
|  | CCCGTCGATTCAGCCAATTG | 60 |
| *CUC 2* | TCCGATTCCACCCAACTGAC | 60 |
|  | ATACCATTCCTTTTCGCCCAT | 60 |
| *CUC 3* | ACCGGAAATACGCCACT | 60 |
|  | ACCCGTCTTTATCCCGTTCG | 60 |
| *PIN10* | CCTTACTCCATGAACCTCCG | 60 |
|  | AACCGTTCTTGCTGACACT | 60 |
| *IAA1* | ATCGCCTTCTCTGTCTTCGT | 60 |
|  | GAAAACCCTCCGCCGCAAA | 60 |
| *gibberellin 20-oxidase* | ACCAGCCACCCACCATGTCG | 60 |
|  | CCCTTCCGTTGCTCCATGCC | 60 |
| *histidine kinase 3* | AATTCCCACACGATCGAGA | 60 |
|  | TCACCGCATACGCTACTCC | 60 |
| *β-Actin* | GATGGGCAGGTGATCACGAT | 60 |
|  | TCTCATGGATTCCAGCAGCT | 60 |
