# Supplementary material for: The Analysis of Genes and Phytohormone Metabolic Pathways Associated with Leaf Shape Development in Liriodendron chinense via De Novo Transcriptome Sequencing
Source: Genes (Basel). 2018 Nov 27;9(12):577. doi: 10.3390/genes9120577 (PMC6316054; doi:10.3390/genes9120577)
Supplement: Supplementary file 1 [file genes-09-00577-s001.zip › 10.genes-366719-supplementary/Table S2. Annotation of candidate genes related to leaf development.docx]

**Table S2.** Annotation of candidate genes related to leaf development

| Description | Annotation name | Regulation | log2Ratio(Lcp7/Lcp2) | Lcp2 FPKM | Lcp7 FPKM | Length |
| --- | --- | --- | --- | --- | --- | --- |
| Homeodomain protein | homeobox protein knotted-1-like 1 | down | -1.8559 | 2348 | 592.5 | 1617 |
|  | homeobox protein knotted-1-like 2 | down | -17.0237 | 142.5 | 0 | 1284 |
|  | homeobox protein knotted-1-like 3 | up | 1.8282 | 63 | 211 | 2182 |
|  | homeobox protein knotted-1-like 6 | down | -3.5092 | 384 | 32 | 1032 |
| Protein containing the NAC DNA binding domain | CUP-SHAPED COTYLEDON 2 | down | -1.4518 | 2118.5 | 711 | 3110 |
|  | CUP-SHAPED COTYLEDON 3 | up | 7.0392 | 1.5 | 198.5 | 993 |
| Transmembrane protein | PIN10 | up | 1.4755 | 934.5 | 2364.5 | 2427 |
| Signal hormonal transduction pathway factor | IAA1 | up | 2.6340 | 360 | 2092.5 | 956 |
|  | gibberellin 20-oxidase | down | -3.2562 | 37.5 | 3.5 | 650 |
|  | histidine kinase 3 | up | 1.2016 | 372 | 791 | 3925 |
