# Supplementary material for: The Analysis of Genes and Phytohormone Metabolic Pathways Associated with Leaf Shape Development in Liriodendron chinense via De Novo Transcriptome Sequencing
Source: Genes (Basel). 2018 Nov 27;9(12):577. doi: 10.3390/genes9120577 (PMC6316054; doi:10.3390/genes9120577)
Supplement: Supplementary file 1 [file genes-09-00577-s001.zip › 10.genes-366719-supplementary/Table S4 The abbreviations.docx]

**Table S4** The abbreviations in text

| Abbreviations | Full name |
| --- | --- |
| SEM | Scanning electron micrograph |
| DEGs | Differentially expressed genes |
| FAA | Formalin–acetic acid–alcohol |
| SAM | Shoot apical meristem |
| TF | Transcription factor |
| *BOP* | *BLADE ON PETIOLE* |
| *KNOX1* | *KNOTTED1-LIKE HOMEOBOX1* |
| *CUC2* | *CUP-SHAPED COTYLEDON2* |
| *PIN1* | *PIN-FORMD1* |
| *HK3* | *HISTINDINE KINASE3* |
| *IPT7* | *ISOPENTENYL TRANSFEREASE7* |
| *STM* | *SHOOTMERISTEMLESS* |
| *BP* | *BREVIPEDICELLUS* |
| *KNAT1* | *KNOTTED-LIKE FROM ARABIDOPSIS THALIANA1* |
| IAA | Indole-3-acetic acid |
| GA | Gibberellin |
| CK | Cytokinin |
| Nr | NCBI non-redundant protein sequences |
| Nt | NCBI non-redundant nucleotide sequences |
| Swiss-Prot | A manually annotated and reviewed protein sequence database |
| COG | Clusters of orthologous groups of proteins |
| KEGG | Kyoto encyclopedia of genes and genomes |
| GO | Gene ontology |
| KO | KEGG ortholog database |
| HV | High Voltage |
| mag | Magnification |
| WD | Working distance |
| det | Detector |
| ETD | Everhart Thonrley Detector |
| SE | Secondary Electron |
| CRE1 | Cytokinin Receptor1 |
| B-ARR | Type-B Arabidopsis thaliana Response Regulator |
| NPR1 | Natriuretic Peptide Receptor |
